# Pseudoxanthoma Elasticum – Also a Lung Disease? The Respiratory Affection of Patients with Pseudoxanthoma Elasticum

**DOI:** 10.1371/journal.pone.0162337

**Published:** 2016-09-13

**Authors:** Simon Pingel, Sebastian Gorgonius Passon, Kristin Solveig Pausewang, Anna Katharina Blatzheim, Carmen Pizarro, Izabela Tuleta, Martin Gliem, Peter Charbel Issa, Nadjib Schahab, Georg Nickenig, Dirk Skowasch, Christian Alexander Schaefer

**Affiliations:** 1 Department of Internal Medicine II, University of Bonn, Bonn, North-Rhine-Westphalia, Germany; 2 Department of Ophthalmology, University of Bonn, Bonn, North-Rhine-Westphalia, Germany; Hospital for Sick Children, CANADA

## Abstract

**Background:**

Pseudoxanthoma elasticum (PXE) is an autosomal-recessive mineralisation disorder caused by loss of function mutations in the ABCC6 Gen. Histological findings and data of an autopsy of a PXE-patient suggest a possible pulmonal calcification. So far, there exists no clinical data whether PXE patients actually are at high risk of developing pulmonary disorder.

**Methods:**

In a cross-sectional study, 35 PXE patients and 15 healthy controls underwent a pulmonary function testing, including spirometry, body plethysmography and carbon monoxide diffusing test. Additionally, PXE patients completed a COPD–Assessment-Test (CAT).

**Results:**

We observed in PXE patients normal values for predicted vital capacity (VC%; 96.0±13.0%), predicted total lung capacity (TLC%; 98.2±12.0%) and predicted forced expiration volume (FEV1%; 102.5±15.6%), whereas compared to healthy controls the PXE group showed significant diminished values for carbon monoxide diffusing capacity (DLCO, 7.2 ±1.4mmol/min/kPa vs. 8.6 ±1.5 mmol/min/kPa; p = 0.008) and predicted carbon monoxide diffusing capacity (DLCO%; 79.7±11.5% vs. 87.2±6.6%; p = 0.008). 11/35 (31.4%) PXE patients showed pathological DLCO% values under 75% (68.5%±5.4%).

**Conclusion:**

PXE patients demonstrated a regular lung function testing, but nevertheless they had impaired CO diffusing parameters, which might be associated with a preclinical state of an interstitial lung disease and a risk for restrictive ventilation disorders.

## Introduction

Pseudoxanthoma elasticum (PXE), also known as Grönblad Strandberg syndrome, is a rare disease with an estimated prevalence of prevalence of 1:25 000 to 1:100 000 [1]. PXE is an autosomal recessive mineralization disorder [2] caused by several loss of function mutations [3] in the ABCC6 gene. It encodes a transmembrane ATP-binding cassette transporter[2] on the basolateral surface [4], mainly expressed in the liver [5], which mediates the cellular release of ATP [2] in healthy people. The released ATP is converted into AMP and anorganic pyrophosphat (PPi) within the liver vasculature [2]. In patients with PXE, lower levels of PPi, which is discussed to be an mineralization inhibitor[6], were observed [2]. Histologic samples from patients with PXE showed thickening and calcification of Bruch membrane [1], extracellular calcification around elastic fibers of the carotid arteries [5] and other vessels, as well as mineralisation and fragmentation of mid-dermal elastic fibers [7].

For example mineralisation can affect eyes, skin or the cardiovascular system [7]. Typical symptoms of skin manifestations are peau d’orange, yellowish papules and inelastic skinfolds, which primarily deteriorate the flexural areas of the body [8]. Common ocular signs of PXE are angoid streaks, retinal pigment epithelium atrophy and characteristic fundus signs [9]. The cardiovascular findings comprise an impairment of the elastic properties of the aorta, a higher prevalence of peripheral artery disease and intermittent claudication [8,10]. Also renovascular hypertension might be a finding in PXE patients [11].

As a systemic mineralization disorder PXE affects elastic fibers [4]. Therefore, it is reasonable to suggest a possible lung affection with increased rate of restrictive ventilation disorder. The aim of our study was to screen for interstitial pulmonary disorder, connected with diminished pulmonary function parameters.

## Material and Methods

In this prospective registry, 35 consecutive PXE patients underwent pulmonary examinations at the Department for Internal Medicine II (cardiology, angiology and pneumology) of the University Hospital Bonn between October 2014 and October 2015. All patients gave written consent and the study was approved by the local ethic committee of the faculty of medicine, Rheinische Friedrich-Wilhelm Universität Bonn.

Minimal criteria for the diagnosis of PXE were the finding of two major clinical signs of PXE like ophthalmological signs (angioid streaks) or characteristic skin lesion [12] or one clinical sign and two mutations in the ABCC6 gene [11]. All patients showed typical ophthalmological signs, 30 patients had a genetically verified evidence of PXE and 26 had skin manifestations. As control group 15 healthy volunteers underwent the same set of examination.

The healthy volunteers had no respiratory diseases and a similar distribution for age, weight, height and smoking, as shown in Table 1.

**Table 1:** Baseline characteristics.

		PXE (N = 35)	Control (N = 15)	p-Value
Age (years)		52.0±9.0	52.2±13.6	0.951
Body Mass Index (kg/m²)		27.8±6.6	25.8±4.9	0.223
Sex (male) %		34.3	53.3	0.208
Smoking	Never %	60.0	40.0	0.193
	Current Smoker %	11.4	6.7	0.607
	Former %	28.6	53.3	0.095
Lung disease	total %	8.6	0.0	0.242
	Asthma %	8.6	0.0	0.242
	COPD %	0.0	0.0	-
	chron. Bronchitis %	2.9	0.0	0.508
Diabetes %		0.0	0.0	-
Hypertension %		37.1	21.4	0.289
Lipid disorder %		42.9	23.1	0.208
Coronary artery disease %		8.6	0.0	0.268
Peripheral artery disease %		77.1	20.0	0.000

### Pulmonary Function Test

All patients underwent a pulmonary function test with spirometry (Flowscreen Jaeger©), body plethysmography (Bodyplethismograph Jaeger©) and a carbon monoxide (CO) diffusing test of the lung in single breath method (Alveo-Diffusionstest Jaeger©).

Standard spirometry (vital capacity (VC), forced expiratory volume (FEV1)), body plethysmography (total lung capacity (TLC), residual volume (RV)) and diffusing parameters (DLCO, DLCO/VA) were recorded. The predicted values for all volumes (TLC%, VC%, RV%, FEV1%) and the diffusion parameters (DLCO% and DLCO/VA%) were calculated automatically by the software of the pulmonary function test, using age, sex, height and weight. Reference parameters were compared according to Global Lung Initiative (GLI) reference values for tiffenau index (FEV1/IVC) and inspiratory vital capacity (IVC). Z-scores were calculated post examination for each patient with the GLI2012 tool (www.lungfunction.org). Diffusing parameters below 75% of the predicted values were assumed as abnormal [13].

Additionally, PXE patients completed a COPD-Assessment-Test (CAT) for quality of life investigation (http://www.catestonline.org/english/index_German.htm) and hemoglobin (Hb) was recorded from laboratory testing at their general practitioner not older than 3 months to eliminate changes in lung function testing caused by anemia. None of our patients had signs of anemia and the mean Hb was 14.17±1.62 mg/dl.

### Statistical Analysis

Presented values were expressed as mean ± standard deviation and a significant P-value was defined as <0.05. For distribution analysis of non-parametric variable Chi² test was applied while differences in parametric variables were tested with a t-test in case of an existing Gaussian distribution, whereas the Mann-Whitney-U-Test was applied in non-Gaussian distributions. Pearsons test was used for parametric correlations, whereas Spearman-Rho test was used for non-parametric correlations. Statistical analysis was performed with SPSS 23 for Windows© (SPSS Inc. Chicago,IL, USA).

## Results

Overall we observed normal values for VC% (96.0±13.0%), TLC% (98.2±12.0%) and FEV1% (102.5±15.6%) in Patients with PXE. None of our patients nor the controls showed FEV1/IVC under the Lower-Limit-of-Normal (LLN = Z-Score < -1.645).

**Fig 1:**
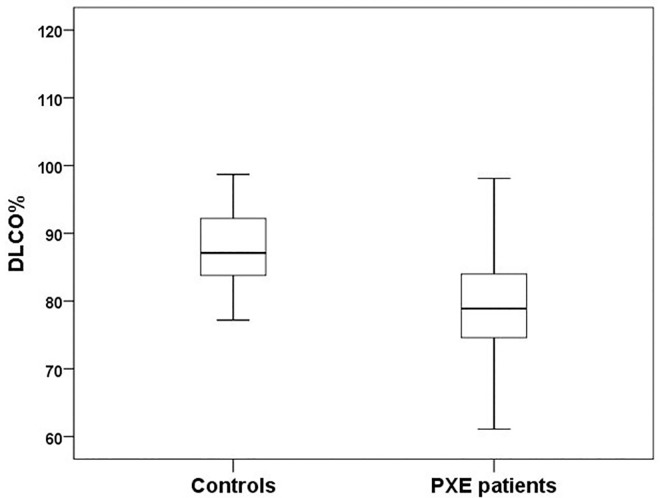
CO diffusion in Comparison. Comparison of predicted CO diffusing capacity (DLCO%) and ratio of CO diffusing transfer coefficient (DLCO/VA%) between PXE patients and healthy controls. DLCO% (79.7%±11.5 vs. 87.2%±6.6; p = 0.008).

As a main finding we observed a significant lower DLCO (7.2±1.4 mmol/min/kPa vs. 8.4±1.5 mmol/min/kPa; p = 0.007) and DLCO% (79.7±11.5% vs. 86.5±6.4%; p = 0.006) compared with the control group (Fig 1). Hence, 11/35 (31.4%) of PXE patients showed a pathological DLCO% under 75% and 2/35 (5.7%) had a DLCO/VA% under 75%.

Additionally, absolute TLC was also significant decreased (5.7 ±1.0 L vs. 6.5 ±1.2 L; p = 0.034) in the PXE group, but only one patient presented a manifest restrictive ventilation disorder with a TLC% < 80%, a VC under the fifth percentile. Other five PXE patients presented a pathological VC under the LLN. Three in combination with a DLCO% below 75%, two with DLCO% values at 75.1% and 76.4%, respectively. [Table 2]

**Table 2:** Repiratory function parameters, for all PXE patients and healthy controls.

	PXE (n = 35)	Control (n = 15)	p-Value
TLC (L)	5.7±1.0	6.5±1.2	0.034
VC (L)	3.6±0.7	4.1±1.0	0.112
RV (L)	2.2±0.5	2.5±0.6	0.090
FEV1 (L)	3.0±0.6	3.4±0.8	0.097
FEV1VC (%)	86.8±6.2	85.5±6.8	0.542
R tot (kPa*s/L)	0.23±0.09	0.18±0.08	0.088
DLCO (mmol/min/kPa)	7.2±1.4	8.4±1.5	0.007
DLCO/VA (mmol/min/kPa/L)	1.4±0.2	1.5±0.1	0.215
TLC%	98.2±12.0	102.3±12.5	0.280
VC%	96.0±13.0	97.9±13.2	0.641
RV%	109.5±25.2	117.6±27.9	0.318
FEV1%	102.5±15.6	103.7±11.6	0.784
FEV1VC%	109.3±9.0	108.9±8.0	0.876
R tot%	77.6±29.6	60.4±25.6	0.099
DLCO%	79.7±11.5	86.5±6.4	0.006
DLCO/VA%	91.2±12.3	98.1±12.4	0.074
DLCO <75% N (%)	11 (31.4)	0.0	0.021
DLCO/VA <75% N (%)	2 (5.7)	0.0	0.379
FEV1VC < LLN (%)	0.0	0.0	-

Repiratory function parameters, for all PXE patients and healthy controls. (TLC = Total Lung Capacity; VC = Vital Capacity; RV = Residual Volume; FEV1 = Forced Expiratory Volume in 1 second; FEV1VC = tiffenau index; R tot = Resistence; DLCO = CO diffusing capacity; DLCO CO diffusing transfer coefficient)

**Fig 2:**
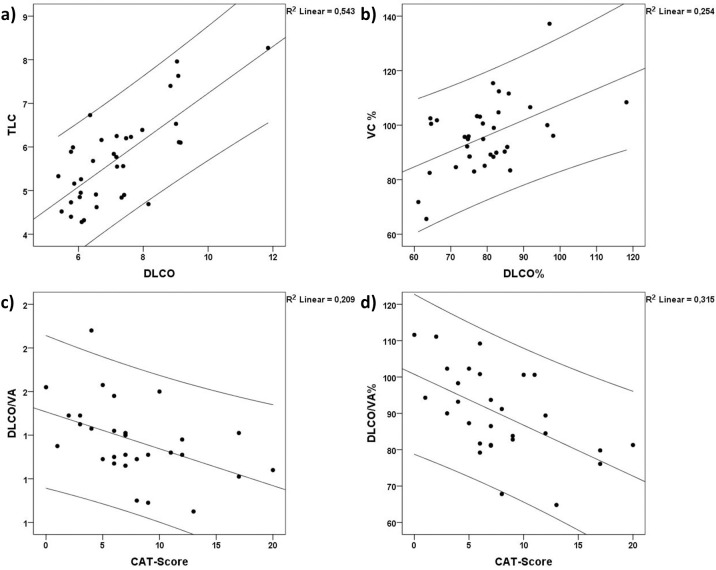
Correlation analysis. Correlations within the PXE patients for Total Lung Capacity (TLC), Vital Capacity (VC), CO diffusing capacity (DLCO), CO diffusing transfer coefficient (DLCO/VA) and CAT-Score. a) Correlation between TLC and DLCO (r = 0.737; p<0.001) b) Correlation between VC% and DLCO% (r = 0.819: p<0.001) c) Correlation between DLCO/VA and CAT-Score (-0.566; p<0.01) d) Correlation between DLCO/VA% and CAT-Score (r = -0.611; p <0.001).

Within the PXE group we performed a subgroup analysis, defined by DLCO% <75% (group1, N = 11, 31.4%) and DLCO% >75% (group2, N = 24, 68.6%). There were no differences in the baseline characteristics [Table 3], but as expected DLCO (6.3 ±0.8 mmol/min/kPa vs. 7.6 ±1.5 mmol/min/kPa; p = 0.009), DLCO% (68.5±5.4% vs. 84.9±9.7%; p<0.001) and DLCO/VA% (82.8±10.4% vs. 95.0±11.4%; p<0.01) were diminished significantly in group 1. In comparison DLCO/VA (1.3±0.2 mmol/min/kPa /L vs. 1.5±0.2 mmol/min/kPa /L; p = 0.062) and VC% (89.8±12.3% vs. 98.8±12.6%; p = 0.06) were decreased, but did not reach significance level. [Table 4]. The statistical analysis revealed high significant correlations between DLCO and TLC (r = 0.737; p<0.001), DLCO and VC (r = 0.819: p<0.001), as well as between VC% and DLCO% (r = 0.504; p = 0.002) (Fig 2A and 2B).

**Table 3:** Baseline Charcteristics in Subgroup Analysis.

		Group 1 (n = 11)	Group 2 (n = 24)	p-Value
Age (years)		49.6±11.3	53.1±7.8	0.375
Body Mass Index (kg/m²)		25.4±5.5	28.94±6.8	0.115
Sex (male) (%)		27.3	37.5	0.554
Smoking	Never (%)	54.5	62.5	0.656
	Smoker (%)	9.1	12.5	0.769
	Former (%)	36.4	25.0	0.490
Lung disease	total (%)	9.1	8.3	0.941
	Asthma (%)	9.1	8.3	0.941
	COPD (%)	0.0	0.0	-
	chron. Bronchitis (%)	0.0	4.2	0.492
Diabetes %		0.0	0.0	-
Hypertension %		0.0	54.2	0.002
Lipid disorder %		27.3	50.0	0.207
Coronary artery disease %		0.0	12.5	0.210
Peripheral artery disease %		72.7	79.2	0.674

Baseline Comparison between PXE patients with diffusing disorder (Group 1) and without diffusing disorder (Group 2). The diffusing disorder were determined as DLCO% under 75%.

**Table 4:** Respiratory function parameters in the subgroup analysis.

	Group 1 (n = 11)	Group 2 (n = 24)	p-Value
TLC (L)	5.6±0.8	5.8±1.2	0.609
VC (L)	3.4±0.6	3.6±0.8	0.358
RV (L)	2.2±0.6	2.1±0.5	0.729
FEV1 (L)	3.0±0.5	3.1±0.6	0.644
FEV1VC (%)	88.9±5.9	85.8±6.3	0.167
R tot (kPa*s/L)	0.23±0.08	0.24±0.10	0.791
DLCO (mmol/min/kPa)	6.3±0.8	7.6±1.5	0.009
DLCO/VA (mmol/min/kPa/L)	1.3±0.2	1.5±0.2	0.062
TLC% (%)	95.9±13.0	99.2±11.7	0.475
VC% (%)	89.8±12.3	98.8±12.6	0.060
RV% (%)	114.1±33.9	107.3±20.7	0.551
FEV1% (%)	96.9±13.6	105.0±16.0	0.136
FEV1VC% (%)	112.3±7.2	107.9±9.6	0.149
R tot% (%)	75.5±25.1	78.51±32.0	0.765
DLCO% (%)	68.5±5.4	84.9±9.7	0.000
DLCO/VA% (%)	82.8±10.4	94.96±11.4	0.005

The mean CAT Score was 7.8± 4.8 in patients with PXE. Group 1 with a DLCO% < 75% had a mean CAT Score of 9.2 ± 5.7 whereas group 2 had a mean CAT Score of 6.9± 4.1 (p = 0.19). The CAT-Score correlated negatively with DLCO/VA (-0.566; p = 0.001) and DLCO/VA% (r = -0.611; p <0.001) (Fig 2C) and 2D)).

## Discussion

According to our knowledge this is the first study investigating a pulmonary affection of patients suffering from PXE. PXE patients had normal lung volumes and no signs for obstructive disorders. In 31.4% of our patients we found pathologically decreased values for DLCO, a very sensitive marker for interstitial lung diseases, and a significant lower TLC. Additionally, one patient with a manifest restrictive ventilation disorder with abnormal decreased TLC and VC was found. Other five patients had also pathological values for VC under the LLN. All patients with low VC presented a DLCO under 75% of the predicted value or were nearby the pathological limit. These functional respiratory values correlated strongly with subjective pulmonary impairment shown by the CAT-score as assessment test for respiratory limitation.

Jackson et al. described a PXE-patient with histological findings of extensive calcification, connected with elastic tissue damage in the lung [14]. In 1996 Yamato et al. published a case report of a lung biopsy with exercise depending dyspnoea in one PXE patient. They found small calcified nodules scattered in the alveolar septa[15]. An autopsy report from Miki et al. demonstrated also vascular changes in the lung of a PXE patients with fragmented, laminated and calcified elastic laminae of the small up to medium size arteries[16].

Reported histomorphological changes in absence of anaemia could be the underlying process in PXE patients leading in a diminished CO diffusing capacity and the trend to restrictive ventilation disorder. Alternatively, interstitial lung involvement could be due to the mineralisation process of the PXE-disease. On the one hand, it could affect the pulmonary tissues directly. On the other hand, damaging the small to medium-sized arteries in the lung could represent another possible pathway.

To reveal other risk factors for a development of pulmonary impairment, we performed a subgroup analysis, including all patients with a DLCO% <75%. This analysis showed no significant differences in the baseline characteristics, therefore identifying independent risk factors other than PXE for a restrictive ventilation disorder in this collective so far could not be found. Further investigations with larger patient cohorts should be performed to address this question. Regarding the rare prevalence recruiting a patient collective with eligible size might be challenging.

## Conclusion

PXE patients had no impairment in spirometry or body plethysmography compared with healthy controls, thus there is no sign for a clinical manifestation of a lung disease. Nevertheless, we found a significant reduction in diffusing parameters, a sensitive marker for preclinical restrictive lung disorders. To our knowledge this is the first time a structured lung assessment of a relatively large cohort of PXE-patients was performed, and it is the first time reporting that PXE patients have unremarkable lung function testing findings, but they are possibly at risk of developing a restrictive ventilation disorder.
